# *In vitro *cultures of circulating tumor cells: a potential tool to unravel drug sensitivity

**DOI:** 10.20517/cdr.2021.121

**Published:** 2022-03-16

**Authors:** Gianluigi De Renzi, Giulia De Marco, Michela De Meo, Eleonora Del Rosso, Paola Gazzaniga, Chiara Nicolazzo

**Affiliations:** Cancer Liquid Biopsy Unit, Department of Molecular Medicine, Sapienza University of Rome, Rome 00161, Italy.

**Keywords:** Liquid biopsy, circulating tumor cells, liquid tumor biomarkers, cell cultures, circulating tumor cell cultures, biomarker evaluation, precision medicine, drug sensitivity

## Abstract

Since taking part as leading actors in driving the metastatic process, circulating tumor cells (CTCs) have displayed a wide range of potential applications in the cancer-related research field. Besides their well-proved prognostic value, the role of CTCs in both predictive and diagnostics terms might be extremely informative about cancer properties and therefore highly helpful in the clinical decision-making process. Unfortunately, CTCs are scarcely released in the blood circulation and their counts vary a lot among different types of cancer, therefore CTC detection and consequent characterization are still highly challenging. In this context, *in vitro *CTC cultures could potentially offer a great opportunity to expand the number of tumor cells isolated at different stages of the disease and thus simplify the analysis of their biological and molecular features, allowing a deeper comprehension of the nature of neoplastic diseases. The aim of this review is to highlight the main attempts to establish *in vitro *CTC cultures from patients harboring different tumor types in order to highlight how powerful this practice could be, especially in optimizing the therapeutic strategies available in clinical practice and potentially preventing or contrasting the development of treatment resistance.

## CIRCULATING TUMOR CELLS: WEAKNESS IN RARITY

During the last decades, personalized medicine has progressively gained a crucial role in cancer therapeutics, leading to an increased need to monitor the molecular heterogeneity of neoplastic diseases. Despite representing the gold standard in defining tumor molecular profile and therefore in guiding treatment choices, tissue biopsy is an invasive practice that does not allow to follow the clonal evolution of the tumor, thus being not very informative of the genomic changes that cancer might exhibit at progressive disease^[1]^. In this context, the increasing interest showed in liquid biopsies together with the consistent number of studies in this research field have led to a deeper consciousness of the potential impact that liquid tumor biomarkers could have on the clinical decision-making process, especially considering that liquid biopsies are non-invasive, easy to perform, and highly accessible^[1,2]^. As the only requirement to obtain information about the tumor mutational status is a simple withdrawal of biological fluids, most frequently blood but also saliva, urine, pleural, and cerebrospinal fluid, liquid biopsies enable a real-time closer look at cancer dynamics, ensuring a more precise monitoring of cancer patients, hopefully offering the best therapeutic option possible^[3]^.

Circulating tumor cells (CTCs), circulating tumor DNA, and more recently exosomes released from both primary tumor and metastatic sites in the systemic circulation represent the main cancer-derived material studied in liquid biopsy-based analyses, providing prognostic and predictive indications and currently under investigation for their potential role in cancer diagnostics^[1,3]^.

CTCs were firstly described by Ashworth^[4] ^in 1869 and, after only twenty years, Stephan Page proposed the “seed and soil” hypothesis, assuming that the metastatic process is not randomly successful, but tumor cells (the “seed”), dethatching from the primary tumor, can grow up in selected organs solely in the presence of a suitable microenvironment (the “soil”)^[5]^. This theory was not immediately accepted, but only after a century, CTCs were recognized for their relevant role in driving the metastasization^[6]^. CTCs, shed actively or passively in the bloodstream, can be found as single cells or aggregated in clusters, with clusters appearing to show up to 50-fold increased metastatic potential compared to single CTCs^[7,8]^. The number of CTCs detected through the antigen-dependent CellSearch® system shows a prognostic value for metastatic breast, colon, and prostate cancers^[9-12]^. CTCs counts ranging from 0 up to 4 CTCs in 7.5 mL of blood are indicative of a favorable prognosis, while a cut-off of 5 CTCs in 7.5 mL of whole blood indicates an unfavorable prognosis for both metastatic breast and metastatic prostate cancers, while in the case of metastatic colon cancer a cut-off of 3 CTCs suggests poor prognosis^[9-11]^. Currently, CTC enumeration through CellSearch® for these three metastatic cancer settings is the only clinical application for CTCs^[13]^.

Unfortunately, since CTCs isolation through the CellSearch® platform relies on the expression of the epithelial cell adhesion molecule (EpCAM), which is normally found on the surface of cells of epithelial origin, there may be problems related to CTCs discrimination and detectability because CTCs are actually capable of downregulating epithelial characteristics in favor of a mesenchymal-like phenotype^[7,14,15]^. Morphological and genetic changes determining this phenotypical switch can be identified in the activation of a biological program termed epithelial-mesenchymal transition^[7,16]^. The acquisition of a mesenchymal-like phenotype strongly enhances the migratory and invasive capability of tumor cells, therefore the metastatic competence of these cells is incredibly increased^[16]^. Moreover, this phenotypical plasticity is highly associated with stemness properties as well as drug resistance, and it is mainly responsible for the impossibility of CellSearch® to detect and isolate CTCs^[7,14-16]^.

To overcome this limitation, several alternative devices based on CTCs physical or functional characteristics have been developed over the years^[7]^. However, the use of these methods is limited to research purposes and currently does not find any applications in clinical practice.

A major issue to consider when studying CTCs is their rarity in blood circulation^[3,6,17]^. The scarce formation of CTCs in the body together with their variability in different tumor types represent hard challenges in CTC analysis^[3,6,17]^. In this context, it is particularly clear the potential usefulness of CTC cultures^[17]^. *Ex vivo *expansions of CTCs can possibly enable to obtain a conspicuous number of circulating tumor cells and consequently to study tumors characteristics not only present in the primary site, but also defining the properties of cells able to survive in the blood circulation as well as to form distant metastases and eventually understanding the changes occurring between the first stages of the tumor and the advanced ones, thus implementing the use of liquid biopsies^[18]^. This would be strongly important, for example, in elaborating new therapeutic strategies for cancer patients and concurrently overcoming the problems related to the onset of resistance phenomena^[3,19-23]^. Despite the huge interest in CTC cultures, only a few groups succeeded in establishing CTC cultures, thus confirming the enormous difficulties that still make this practice extremely challenging today^[12]^.

## CTC BIOLOGY

CTCs have a crucial role in tumor progression, taking part in the intermediate stage of the process known as metastatic cascade^[24]^. Indeed, the metastatic evolution of cancer is a sum of several events, starting with tumor cells dissemination from the primary site of the neoplastic mass (or alternatively from an already formed metastatic lesion), followed by a phase of invasion directly into the blood vessels or indirectly passing through the lymphatic circulation, ultimately concluding with the colonization of a distant organ and the new tumor formation^[25,26]^.

CTCs intravasation can occur both through active and passive shedding^[26]^. Cell-intrinsic features, microenvironmental characteristics, and vascular structure could be involved in the generation of CTCs^[26]^. The activation of the epithelial-mesenchymal transition program, guided by a group of different transcriptional factors (e.g., Snail, Slug, Twist, and Zeb1), leads tumor cells to lose cell polarity, acquire the capability to degrade components of the extracellular matrix, and downregulate such epithelial characteristics like EpCAM and E-cadherin surface proteins (extremely important for cell-cell adhesion) in favor of mesenchymal-like features, together with the invadopodia formation regulated by the N-WASP protein, concur to the active release of cancer cells in the bloodstream^[25-27]^. The presence of a leaky vascular structure, highly associated with tumor growth, can contribute to the generation of CTCs^[26]^. The chronical activation of angiogenesis is notably one of the hallmarks of cancer^[28]^. The dysregulation of proangiogenic signals, like the fibroblast growth factor (FGF) and the vascular endothelial growth factor (VEGF), promotes the formation of aberrant vessels, thus facilitating both the passive shedding of cancer cells and the active intravasation^[26,28]^. Lastly, the tumoral microenvironment plays a key role in inducing the formation of CTCs^[26]^. Interestingly, it has been reported that tumor-associated macrophages, expressing the tyrosine kinase with immunoglobulin-like loops and epidermal growth factor homology domains-2 (Tie-2), are capable of producing VEGF, therefore contributing to the increase of vascular permeability and ultimately to tumor cells intravasation^[29]^.

Once in circulation, it is possible to find CTCs both as individual cells and clusters of 2 to 50 cells^[26]^. Several studies regarding CTC clusters detection, their characteristics and functional role have been published during the last decade. Particularly interesting is the work of Aceto *et al.*^[30] ^demonstrating that CTC clusters derive from oligoclonal groups of cells arising from a single tumor, excluding cluster formation by coalescence of single CTCs in the bloodstream. This study ulteriorly reported the half-life of CTC clusters, ranging from 6 to 10 min, thus considerably shorter compared to single CTCs (estimated to be 25 to 30 min)^[30]^. The reduced half-life of CTC clusters, together with the ability to extravasate faster than single CTCs and the enhanced metastatic competency (partly associated with resistance to apoptosis), support their survival and outgrowth^[30]^.

In 1975, Butler and Gullino^[31] ^quantified the rate of tumor cells shedding in the bloodstream using a rat model. This study showed that tumors release millions of cancer cells in the blood circulation per 24 h/g of tissue^[31]^. Despite the huge amount of tumor cells shed each day, patients commonly develop only few metastases, thus demonstrating the metastatic process as highly inefficient^[32]^. Indeed, the cell viability in the blood is compromised by several factors, including shear stress, *anoikis*, the deficit of growth factors, and immune surveillance^[32,33]^. The combination of such aspects results in a tremendously limited number of CTCs, commonly 1 to 10 CTCs per mL of blood^[33,34]^.

Although many factors concur in reducing the survival of CTCs in the circulation, important evidence indicates various blood constituents, including platelets, neutrophils, macrophages, myeloid-derived suppressor cells (MDSCs), or cancer-associated fibroblasts to tightly interact with CTC preserving them from physical damage and helping them in evading the immune system^[35]^. Platelets are actively involved in CTCs’ protection during their transit into the bloodstream in many ways, for example, defending them from mechanical stress, as well as cancer-associated fibroblasts, and inducing resistance from *anoikis*, which is regulated by the YAP pathway, determining RhoA-(myosin phosphatase targeting subunit 1) and MYPT1-protein phosphatase (PP1)-mediated Yes-associated protein 1 (YAP1) dephosphorylation and nuclear translocation^[35,36]^. Most importantly, platelets can recruit either neutrophils and macrophages by the release of chemokines, like CXCL5 or CXCL7^[37]^. Together with neutrophils, platelets can shield tumor cells from other immune cells attacks, thus helping CTCs in immune escaping^[35,37]^. Similarly, CTC-MDSC clusters seem to be capable of evading T cell immune response^[35]^. Moreover, interactions with blood cells can significantly enhance the ability of those CTCs to invade distant sites. It has been reported that platelets can release transforming growth factor β (TGFβ), known to be involved in the activation of the epithelial-mesenchymal transition program, thus incrementing the invasion potential of CTCs, and other mediators, like histamine, serotonin, and eicosanoid metabolites among others, determining the modification of blood vessel permeability^[37-39]^. On the other hand, cancer cells releasing colony-stimulating factor 1 can activate macrophages, which, in turn, secrete epidermal growth factor, therefore inducing tumor cell migration^[40]^.

Complex interactions with blood constituents combined with the expression of specific genetic signatures, which seem to be associated with the ability of CTCs to metastasize (e.g., in a study by Zhang *et al.*^[41]^, breast cancer CTCs expressing the Her2+/EGFR+/HPSE+/Notch1+ genetic signature demonstrated a tendency to metastasize in the brain), allow an extremely small fraction of cancer cells in the bloodstream to complete the metastatic cascade, thus colonizing distant tissues and promoting the generation of new tumor lesions.

Hence, it is clear that CTCs with metastatic competence are definitely rare events, delineating the metastatic cascade as a globally inefficacious process. Furthermore, although it is possible to count a large number of works elucidating the main characteristics of CTCs, there are lots of questions yet unraveled. Futures studies, providing a deeper knowledge of the biology of these circulating cells, are needed to consolidate the clinical value and the use of CTCs, particularly in the field of precision medicine.

## CTC CULTURES: A STRENUOUS CHALLENGE

**Figure 1:**
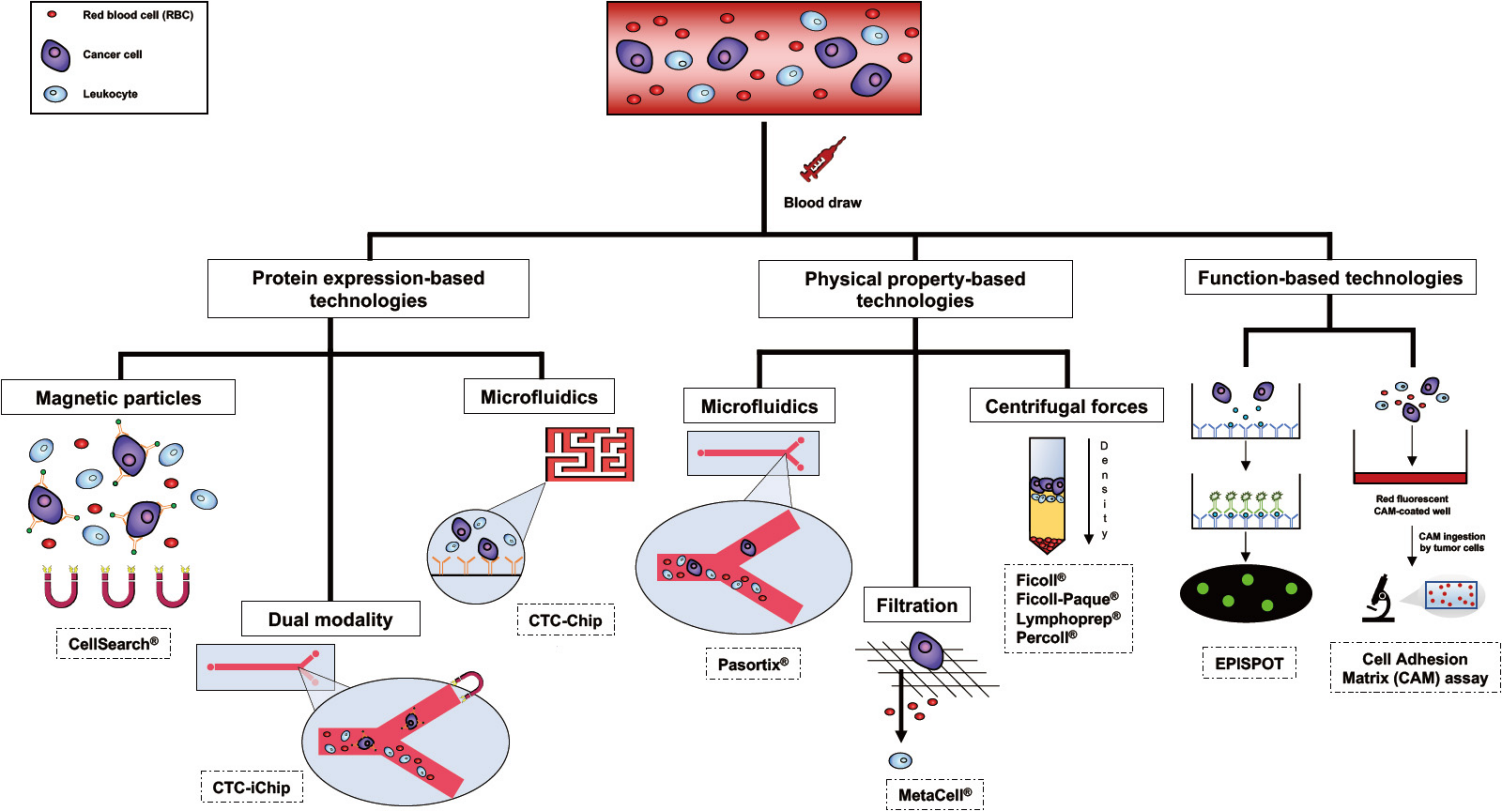
Schematic examples of the most commonly used technologies for circulating tumor cell (CTC) enrichment, detection, and isolation.

Several strategies are currently available for CTCs isolation and subsequent culturing. The enrichment is a crucial step when it comes to isolating viable CTCs from the rest of the blood constituents, such as platelets, red blood cells and white blood cells, allowing CTCs concentration and thus facilitating the detection process^[12,17]^. It is possible to perform the enrichment step through three different types of technologies: protein expression-based, physical property-based and function-based technologies^[12,17] ^[Figure 1].

In the first case, the enrichment feasibility is due to the expression or the lack of the expression of specific proteins on the surface of CTCs. In other terms, CTC isolation relies on the presence of certain positive or negative selection markers^[12,17]^. EpCAM is one of the most frequently used surface proteins for positive enrichment, while surface antigens, like CD45, serve to guarantee the depletion of leukocytes from the blood sample by negative selection^[12,17]^. Among the positive selection-based techniques, immune-magnetic separation, flow cytometry and immuno-affinity based microfluidic platforms, like the CTC-iChip, must be mentioned^[12]^. The drawback of these methods is strictly related to the possible loss of CTCs during the isolation, due to the lack of expression of the antigens selected for the positive enrichment^[12,17]^. To overcome this problem, CTCs can alternatively be separated by negative selection. RosetteSep® antibody cocktail, for example, is an available product currently used to enrich CTCs through tetrameric complexes of antibodies that target the unwanted cells in the blood sample, allowing their removal and eventually CTC purification^[12]^. These tetrameric antibody complexes recognize several antigens, including CD2, CD16, CD19, CD36, CD38, CD45, CD66b, and glycophorin A on red blood cells and leukocytes^[42]^. After centrifugation using a density gradient medium, purified tumor cells can be found at the interface between the plasma and the medium^[42]^.

Since CTCs are supposed to be larger and less deformable than other blood cells (even though recent studies showed the existence of small CTCs potentially implicated in metastasis progression) and have different relative densities compared to red blood cells and white blood cells^[43]^, separation based on the physical properties play a leading role in the field of CTC enrichment^[12,17]^. Indeed, it has been generally reported that CTCs exhibit an average size of 30 μm, bigger than red blood cells (6-8 μm) and white blood cells (10-15 μm)^[44,45]^. Thus, several size-based platforms have been developed to isolate CTCs, such as MetaCell®, as well as different microfluidic devices, like the Parsortix®^[12]^. While MetaCell® enables CTC enrichment through an 8 μm-pore polycarbonate membrane, Parsortix® is a microchip-based isolation technique^[46-50]^. Both methods give the possibility to isolate viable CTCs^[12]^, with MetaCell® also being frequently used to establish short-term CTC cultures^[46-50]^. Moreover, in 1959, Seal, observing that red blood cells and white blood cells have higher specific gravities (1.092 and 1.065, respectively) compared to cancer cells (1.056), exploited these differences to separate them using one of the first density-based techniques involving silicone floatation^[51]^. To date, it is possible to count on different density gradient media, such as Ficoll®, a synthetic polymer formed by copolymerization of sucrose and epichlorohydrin^[12]^, Ficoll-Paque® and Lymphoprep®, both composed of polysaccharides and diatrizoate^[52]^, and Percoll®, consisting in a colloidal silica particle suspension^[45]^. All these media basically allow the separation of CTCs from blood cells through centrifugation. To optimize this type of CTC enrichment, density gradient media are frequently used with other methods, like the aforementioned RosetteSep® antibody cocktail^[53]^.

Eventually, another opportunity to enrich viable CTCs is offered by the isolation of tumor cells based on their functional properties^[12,17]^. Almost ten years ago, the development of a method allowing CTCs enrichment by their invasion ability, known as collagen adhesion matrix assay, represented the first example of this methodological category^[17]^. Essentially, this assay is based on the singular propension of tumor cells to disrupt and ingest collagen adhesion matrix fragments, providing information about cancer cells invasiveness^[54]^. On the other hand, the detection of proteins secreted, released or shed by viable cancer cells, is the core of the EPithelial ImmunoSPOT (EPISPOT) assay^[55]^. These types of techniques enable *in vitro *CTC expansions since they well preserve cell viability^[12,17] ^[Table 1].

**Table 1:** Examples of strategies for viable circulating tumor cell (CTC) isolation

**Type of technology**	**System**	**Method**	**Type of culture**	**Ref.**
Protein expression-based technologies	RosetteSep®	Antibody cocktail for negative selection	Short-term Long-term	Guo *et al*.^[12]^
	CTC-iChip	Micro fluidic capture platform with two immune magnetic sorting modes to isolate CTCs	Long-term	Yu *et al*.^[19]^
Physical property-based technologies	Ficoll® Ficoll-Pacque® Lymphoprep® Percoll®	Density gradient media that allow the separation of circulating tumor cells from blood cells through centrifugation	/	Guo *et al.*^[12]^ Li *et al.*^[45]^ Rosado *et al.*^[52]^
	MetaCell^®^	8 μm-pore polycarbonate membrane-based technique.	Short-term	Kolostova K. *et al*.^[46]^
	Parsortix®	Cell size and deformability microchips isolation based technique	/	Guo *et al*.^[12]^
Function-based technologies	Collagen adhesion matrix assay (CAM)	Method based on tumor cells’ ability to attach and ingest collagen adhesion matrix	/	Guo *et al*.^[12]^
	EPithelial ImmunoSPOT assay (EPISPOT)	Detection of proteins secreted or released by viable cancer cell	Short-term	Guo *et al*.^[12]^
			Long-term	Cayrefourcq *et al*.^[62]^

Despite the numerous methods currently available for CTCs enrichment and therefore the resulting complexity in selecting a more suitable technique to enrich viable CTCs for future cultures, the choice of the culturing conditions is probably the hardest part of the *in vivo *expansion process. In this context, the critical challenge is related to the limited knowledge we have about the biology of the CTCs^[12] ^and, most of all, the prerogative of CTCs to show a high rate of heterogeneity^[14] ^that overcomplicate the adoption of certain culturing options and is consequently decisive in succeeding or failing the *ex vivo *CTC propagation^[12]^. According to the scientific literature, it is possible to count on a discrete number of studies reporting examples of CTCs *in vitro *cultivations. In 2013, Zhang *et al.*^[41] ^reported for the first time an *ex vivo *expansion of CTCs for breast cancer, also demonstrating those CTCs to have metastasis initiating properties in the brain when expressing a specific genetic signature (the aforementioned Her2+/EGFR+/HPSE+/Notch1+). For this study, CTC cultures were monitored over 28 days, therefore it was not possible to establish long-term cultures^[41]^. Comparable cultures of circulating cancer cells were obtained from patients with mesothelioma^[56]^, lung^[57]^, esophageal^[58]^, bladder^[59]^, and head and neck cancers^[60]^. In all these cases, CTCs were efficiently maintained in culture for a short period (in most cases, 14 days, with rare exceptions, which, however, did not exceed 50 days of cultivation)^[56-60]^. Only a few groups successfully obtained long-term CTC cultures, including Yu *et al.*^[19] ^and Gao *et al.*^[61] ^in 2014, respectively for breast and prostate cancers. Additionally, Cayrefourcq *et al.*^[62] ^in 2015, Grillet *et al.*^[21] ^in 2017, and Soler *et al.*^[63]^ in 2018 were able to establish durable CTCs cultures and permanent CTC lines from colorectal cancer patients^[62,63]^. Brungs *et al.*^[22] ^obtained long-term CTC cultures in 2020 for metastatic gastroesophageal cancer, while Hamilton *et al.*^[20] ^in 2015 and Lee *et al.*^[23] ^in 2020 obtained successful CTC cultures from small cell lung cancer (SCLC) patients.

The two main strategies adopted to establish *in vitro *circulating cancer cell cultures are represented by two-dimensional (2D) and three-dimensional (3D) cultures^[12]^. Adherent conditions are the most common choice, especially for short-term cultures, because it is clearly easier to set up a 2D culture, in terms of time, complexity, and costs^[12,64]^. However, even if there are studies reporting data of successful short-term and long-term CTC cultures in both 2D and 3D systems, it has been observed that a non-adherent culturing condition is preferable when the aim is to establish long-term cultures, because following a few cell divisions, CTCs cultured according to a monolayer adherent approach tend to senesce^[19]^. Besides this, when cultured in 2D conditions, CTCs actually lose essential morphological characteristics as well as cell-cell and spatial interactions, thus several cellular functions, like proliferation or differentiation, are definitively compromised^[64]^. Moreover, the adherent culture condition guarantees unlimited access to nutrients and oxygen, in contrast with the actual *in vivo *situation^[64]^. Different studies reported the importance of hypoxic conditions in promoting CTCs growth^[19,62]^, while other publications showed the capability of CTCs to grow even under a non-hypoxic context^[61]^.

Although recreating the exact characteristics of a tumor microenvironment is almost impossible, there have been many successful attempts in culturing CTCs as well as mimicking the *in vivo *tumor growth, for example, co-cultures of CTCs from early lung cancer patients together with tumor-associated fibroblasts, collagen I, and Matrigel^[57]^.

Alternatively, *ex vivo *expansions of CTCs can be implemented by direct inoculation of CTCs in immunodeficient mice. Major brilliant examples of CTC-derived explants were published during the last two decades, starting with the work of Pretlow *et al.*^[65] ^in 2000, where the authors reported the capacity of cells in peripheral blood of prostate and colorectal cancer patients to form metastasis. In 2013, Baccelli *et al.*^[66]^ demonstrated the existence of a population of metastasis-initiating cells among breast cancer CTCs that express EPCAM, CD44, CD47, and MET, thus recapitulating the phenotype of patient metastases. Lastly, Hodgkinson *et al.*^[67] ^elegantly described that CTCs isolated from SCLC patients, either showing sensitivity or resistance to chemotherapy, then inoculated into immune-compromised mice were tumorigenic and mirrored the donor patient’s response to treatments. It is important to point out that the feasibility of these *in vivo *assays is associated with the presence of very high concentrations of CTCs in the blood samples (e.g., > 1000 in 7.5 mL of peripheral blood in the case of breast cancer patients), therefore reducing the possibility to successfully realize CTC-derived explants^[62]^.

However, *ex vivo *propagations of cancer cells are still extremely delicate processes, therefore more and more studies are needed to optimize all the steps involved in this practice.

## CTC MOLECULAR CHARACTERIZATION: DIVING INTO PRECISION MEDICINE

Cancer is definitely not a static disease, therefore spatial and temporal tumor dynamics must be considered when analyzing the neoplastic evolution to guide patients towards the best clinical outcome possible^[68]^. Since treatment decisions solely rely on primary tumor sampling, there clearly could be a massive loss of information about tumor characteristics that would be otherwise important in the therapeutic evaluation process, especially considering that treatments are actually directed against metastases rather than primary tumors^[68,69]^. Indeed, the selective pressure deriving from both the metastatic process and microenvironmental features can induce the formation of subclones presenting properties that highly differ from the primary tumor^[7]^. Thus, the analysis of metastatic cells is crucial for clinical practice purposes^[7]^. However, in several cases (e.g., lung or brain metastases), tissue sampling is certainly not feasible^[7]^. Given that metastatic cells can be constitutive of the pool of CTCs, liquid biopsy can definitely provide a valid alternative to this invasive practice^[69]^.

The molecular characterization of CTCs might offer details about the changes occurring during the metastasization process and possibly elucidate the reasons for resistance to therapy as well as highlight potential therapeutic targets^[69]^. In breast cancer, for example, human epidermal growth factor receptor 2 (HER2) positive CTCs were found in patients with HER2-negative primary tumors suggesting the proper therapeutic regimen based on the HER2-CTC status^[70-72]^. Comparably, patients with estrogen receptor (ER) positive primary tumors were found to be characterized by the presence of ER-negative CTCs^[73]^. This may predict the onset of resistance to endocrine treatment in the subpopulation of metastatic breast cancer patients who do not benefit from this therapy^[7,73]^. Other analyses of CTCs at the single-cell level showed to be informative of the estrogen receptor 1 (*ESR1*) gene mutations, thus allowing the identification of genes involved in endocrine treatment resistance^[74]^. In metastatic castration-resistant prostate cancer (mCRPC), an androgen-dependent type of prostate cancer, the cause of a failing response to androgen receptor (AR) inhibitors, enzalutamide and abiraterone, can be identified in the presence of androgen-receptor splice variants^[75]^. Androgen-receptor splice variant 7 (AR-V7) was actually found to be expressed on the surface of CTCs isolated from mCRPC patients, thus confirming the association between the detection of AR-V7 in CTCs and the exerted resistance to AR inhibitors in mCRPC patients^[75]^. In a study focused on colorectal cancer, genomic analyses conducted on individual CTCs showed the presence of both Kirsten rat sarcoma viral oncogene homolog (KRAS) mutated and KRAS wild-type CTCs in the same patient^[76]^. Since the expression of KRAS mutations hinder the use of anti-epidermal growth factor receptor (EGFR) treatments, the presence of CTCs harboring KRAS mutations in wild-type colon cancers might explain the therapeutic EGFR inhibition failure, thus being indicative of treatment resistance^[7,77,78]^. Lastly, programmed cell death ligand 1 (PD-L1) expression on CTCs in metastatic lung cancer seems to be predictive of a good response to anti-PD-1 immunotherapy, so it could potentially become a helpful biomarker when evaluating the response to immunotherapies in the context of precision medicine^[79]^.

## *IN VITRO* CTC CULTURES AS A MODEL TO DECIPHER DRUG SENSITIVITY

Considering the low concentration of CTCs in the bloodstream as well as their high rates of heterogeneity and poor survival, not surprisingly, there are still a lot of open questions about CTC biology, including the timing of their release in the blood, the extravasation and intravasation processes, their ability to survive once in the systemic circulation and which is the genetical relation with the original tumor. Moreover, despite their identification and study in different neoplastic diseases, the aforementioned issues (first and foremost, the poor number of CTCs released in the blood) represent remarkable obstacles to CTCs characterization at genomic, transcriptomic, and functional levels. Therefore, *in vitro *culturing of CTCs can offer the opportunity to overcome these limitations and better elucidate the molecular features of these cells.

**Figure 2:**
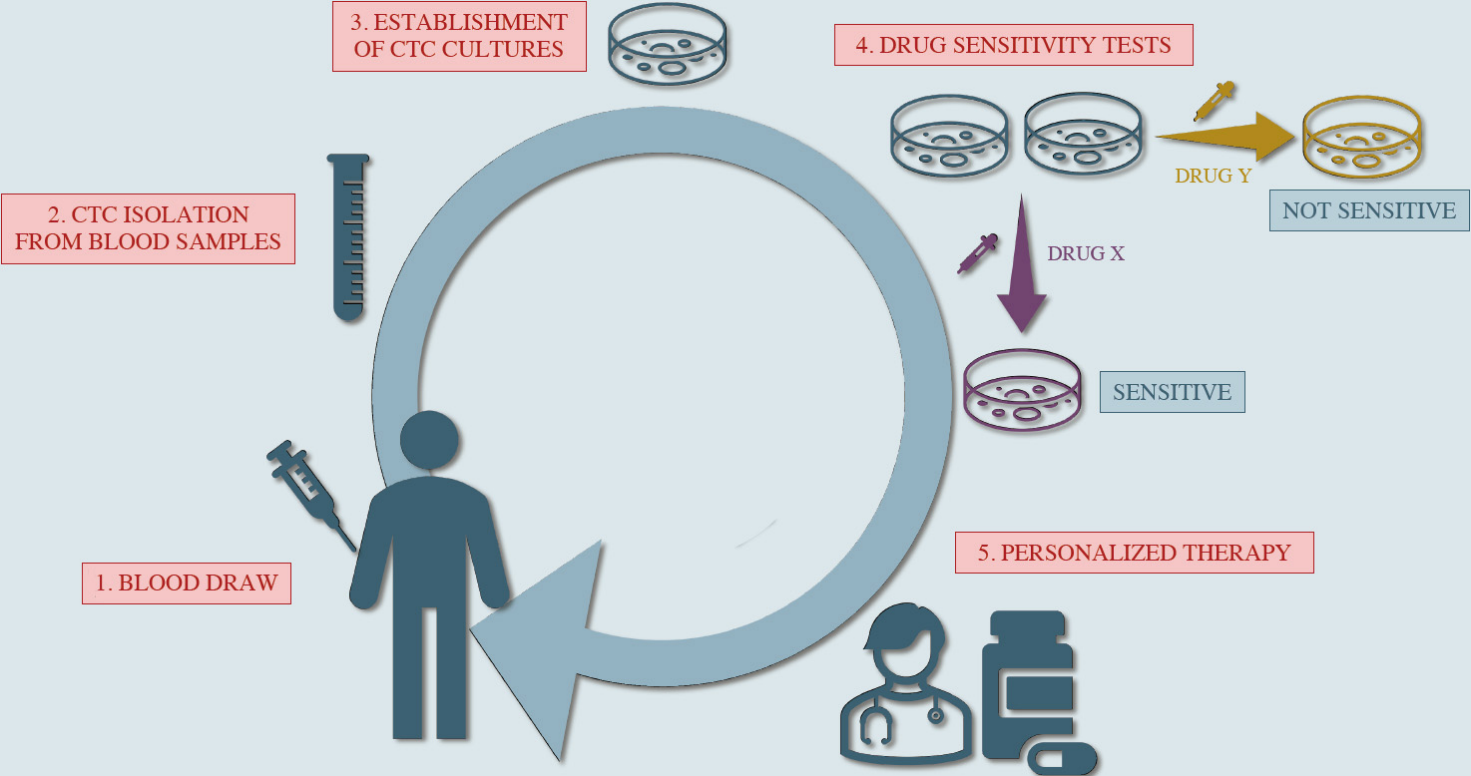
Potential use of circulating tumor cell (CTC) cultures as a predictor for drug sensitivity/resistance, guiding to a more personalized type of therapy.

Unfortunately, CTCs are definitely not easy to expand *in vitro*, especially when the purpose is to establish long-term CTCs cultures^[8,12,80,81]^. However, for those groups who succeeded in culturing CTCs, it was possible to observe that these cell lines they obtained show phenotypical characteristics that partially match those of cells present in tumor tissues from donor patients, but also express molecular features specifically related to CTCs^[19,41,62,82]^, exhibiting metastatic competency^[66,67] ^as well as stemness properties including an efficient DNA repair system and an enhanced metabolic rate^[82]^. Furthermore, since being representative of the tumor molecular landscape, CTC cultures can be useful in predicting drug sensitivity as well as treatment resistance and eventually became a precious tool in drug screening projects^[83] ^[Figure 2].

In a ground-breaking study, dated 2014, Yu *et al.*^[19] ^isolated viable CTCs, using the CTC-iChip technology, and successfully established long-term oligoclonal CTC cultures from six ER-positive breast cancer patients and sustained these cultures for over six months. The analysis of these cell cultures allows exploration of the unique genetic context of single tumors, thus revealing the presence of preexisting mutations in phosphatidylinositol-4,5-bisphosphate 3-kinase (PIK3CA) gene and the acquisition of new tumor-derived mutations in *ESR1 *gene, *PIK3CA *gene, and fibroblast growth factor receptor gene 2 (*FGFR2*), among others^[19]^. Furthermore, the CTC lines obtained were used for drug sensitivity testing, they resulted concordant with patients’ clinical histories, and they were also useful for the identification of new potential therapeutic targets^[19]^. More specifically, one of these cell lines, displaying a high allele frequency of mutant ERS1, was actually not sensitive to selective ER modulators and the selective ER degrader fulvestrant^[19]^. However, since ER strongly depends on the activity of HSP90 protein and the stabilization of mutated receptors relies on this chaperone, the use of HSP90 inhibitor in this ERS1 mutated cell line was found to be cytotoxic alone and in combination with both raloxifene and fulvestrant^[19]^. In another CTC line, harboring both *PIK3CA *and *FGFR2 *gene mutations, the combined inhibition of these two targets (not previously tested in clinical settings) showed to successfully enhance the activity of single drugs^[19] ^[Table 2].

**Table 2:** Patients involved, CTC lines obtained and drug sensitivities

**Authors**	**Type of tumor**	***n *** **patients involved**	***n *** **of CTC cell lines obtained**	**CTC lines name**	**Molecular signatures**	**Sensitivity**
Yu *et al.*^[19]^	Metastatic luminal subtype breast cancers	36	6	BRx-33	*ESR1*, *NUMA1*	/
				BRx-07	*TP53*, *PIK3CA*, *FGFR2*, *CDH1*, *APC*, *DGKQ*, *MAML2*	Paclitaxel*, fulvestrant* and doxorubicin* FGFR2 inhibitor AZD4547 *PIK3CA *inhibitors (BYL719 and PD173074) Moderately responsive to the *FGFR1 *inhibitor PD173074
				BRx-68	*TP53*, *ESR1*, *PIK3CA*, *MSN*	Capecitabine*, fulvestrant* HSP90 inhibitor STA9090 alone and in combination with both raloxifene and fulvestrant
				BRx-50	*ESR1*, *IKZF1*, *BRCA2*	Capecitabine*, olaparib*
				BRx-42	*PIK3CA*, *KRAS*, *IGF1R*	/
				BRx-61	*TP53*	/
Hamilton *et al.*^[20]^	Small cell lung cancer	2	2	BHGc7	/	Cisplatin, etoposide, topotecan and epirubicin
				BHGc10	/	Etoposide, topotecan and epirubicin Mild resistance to cisplatin
Grillet *et al*.^[21]^	Metastatic colorectal cancer	/	3	CTC41	*BRAF V600E*, CSC-related genes (*ALDH1A1*, *CD133*, *CD26*, *CD44v6*)	Resistant to FIRI (regimen inspired by standard-of-care chemotherapy combinations 5-fluorouracil and SN-38, the active metabolite of irinotecan)
				CTC44		
				CTC45		
Brungs *et al.*^[22]^	Metastatic gastroesophageal cancer	20	1	UWG01CTC	Genes for neuroendocrine markers (*CNTFR, PAX-5*, *NGF*), *DLL-3*	Synergism at all concentrations of carboplatin and etoposide Carboplatin increase radiosensitivity
		41	1	UWG02CTC	*Helicobater pylori *mediated carcinogenesis genes (*AKT24*, *ETS225*, *MYC*), *CDH1*, CSC-related genes (*CD44*, *ALDH1*, *CD133*), *EGFR FGFR2*, *HER-2*, *MET*, DNA repair kinases (*ATM *and *ATR*), notch ligand delta-like ligands (*DLL-1 *and *DLL-4*), *PLA2GA*, *JAK/STAT *pathway genes	30-40× more sensitive to doxorubicin and etoposide than UWG01CTC
Lee *et al.*^[23]^	Small cell lung cancer	22	18	/	*TTF-1*, synaptophysin, *PD-L1*, variable expression of EMT markers (E-cadherin, N-cadherin)	CTC lines from patients 14 and 20 showed high sensitivity to standard treatment for SCLC patients (cisplatin/etoposide), whereas these drugs exhibited no cytotoxicity in the CTC line from patient 15, reflecting patient lack of response to this therapy

*Sensitivity to these drugs was consistent with clinical history of the patients. CTC: Circulating tumor cells; EMT: epithelial-mesenchymal transition; FIRI: fluorouracil and irinotecan; FGFR 1 and 2: fibroblast growth factor receptor 1 and 2; HSP90: heat shock protein 90; PIK3CA: phosphatidylinositol-4,5-bisphosphate 3-kinase catalytic subunit alpha.

Hamilton *et al.*^[84]^, who firstly obtained stable CTCs cultures from SCLC patients in 2015, later in the same year conducted a drug sensitivity study using two CTCs lines, BHGc7 and BHGc10, that were established from peripheral blood of SCLC patients with the extended disease^[20]^. Since CTCs counts are monitored for prognostic purposes and to evaluate response to cytotoxic therapy, they treated SCLC BHGc7 and BHGc10 CTC cell lines with common second-line therapies for SCLC (cisplatin, etoposide, topotecan, and epirubicin) *in vitro *and compared the chemosensitivities of these cell lines to drug responsiveness of several permanent SCLC cell lines derived from lung and distinct metastases^[20]^. The cytotoxicity assays showed that BHGc10 was way more resistant than BHGc7 to cisplatin, while the other SCLC cell lines exerted variable responses to this chemotherapeutic agent^[20]^. Both BHGc7 and BHGc10 were sensitive to etoposide as well as two SCLC cell lines^[20]^. The remaining SCLC cell lines displayed elevated IC_50 _values instead^[20]^. Either BHGc7 and BHGc10 were found to be highly sensitive when treated with topotecan or epirubicin^[20]^. Compared to the CTC cell lines, SCLC cell lines exhibited different susceptibilities to epirubicin and topotecan, showing a significant resistance to epirubicin, while milder to topotecan (with the exceptions of topotecan-sensitive SCLC26A cell line and, conversely, NCI-H526 distinctly topotecan-resistant)^[20]^. The heterogeneous responses displayed by CTCs in comparison to primary and metastatic SCLC cells not only highlight the intrinsic difference in terms of drug sensitivities among these cancer cells, but also clearly indicate that the variation in CTCs number can possibly not mirror the response of primary and metastatic SCLC lesions to chemotherapeutic treatment^[20]^ [Table 2].

Later in 2017, Grillet *et al.*^[21] ^established three distinct CTC cell lines by isolating and expanding CTCs from chemotherapy-naïve patients with metastatic colorectal cancer. These cell lines, cultured for several months, showed up to have strong cancer stem cell phenotypical traits, with self-renewal and multilineage differentiation properties as well as metastatic potential and a robust expression of typical cancer stem cell markers, ranging from aldehyde dehydrogenase (ALDH1A1) and CD133 to CD26 and CD144V6^[21]^. Since being genetically heterogeneous is another prerogative of these CTC lines, it has been demonstrated that they surprisingly harbor the BRAF V600E mutation, although the primary tumors as well as metastatic tissues carried KRAS mutations^[21]^. Additional analyses provided data highlighting the differential upregulation of metabolic activity in CTC lines compared to primary tumor-derived cells, with a special regard for the enhanced drug/xenobiotics metabolism, thus suggesting these cells to be strongly resistant to standard cytotoxic compounds^[21]^. These findings were confirmed by following drug sensitivity tests, using a chemotherapy regimen (FIRI: 5-fluorouracil and the active metabolite of irinotecan) that basically mimics the standard treatment combinations for colorectal cancer patients^[21]^. CTC lines demonstrated more resistance to this therapeutic combination than cells derived from both primary and metastatic tumors^[21]^. Lastly, evaluating the multikinase inhibitor regorafenib and the BRAF V600 inhibitor vemurafenib activity on these cell lines, a variable sensitivity to regorafenib was exerted and specifically one of these CTC lines emerged to be responsive to vemurafenib, although this BRAF V600 inhibitor demonstrated a scarce efficacy in BRAF-mutated colorectal cancer patients^[21] ^[Table 2].

More recently, a study by Brungs *et al.*^[22] ^reported the establishment of long-term CTC cultures (maintained for over 12 months), with CTCs isolated from metastatic gastroesophageal cancer patients. Profiling the two CTC lines obtained (UWG01CTC and UWG02CTC), data showed these cell lines to display distinct genotypic and phenotypic features, basically reflecting the characteristics of the originating tumor^[22]^. As the first CTC line (UWG01CTC) was obtained from a patient whose gastrointestinal cancer rapidly developed into metastatic disease, with metastasis histopathology showing high-grade neuroendocrine carcinoma, UWG01CTC exhibited high levels of neuroendocrine markers (i.e., synaptophysin, CD56, and chromogranin A) and strongly expressed genes encoding for neuroendocrine markers such as the ciliary neurotrophic factor receptor (CNTFR), the B-cell-specific activator protein (PAX-5), and the nerve growth factor receptor (NGFR), although this line was negative when stained for epithelial (e.g., EpCAM) or stem cell markers^[22]^. On the other hand, the patient whose CTC sampling generated the UWG02CTC line was affected by a gastric adenocarcinoma, thus robustly expressing cytokeratins (CK), in particular CK-20, as well as EpCAM and E-cadherin proteins, but also gastric cancer stem cell markers CD44, ALDH1, and CD133, and demonstrating stem cell pathways, like NOTCH and WNT, to be upregulated^[22]^. Considering the differences these two CTC lines exhibited, drug and radiosensitivity were evaluated^[22]^. Indeed, UWG01CTC and UWG02CTC responded differently to standard chemotherapeutics used for these cancers^[22]^. Moreover, harboring distinct molecular landscapes, with UWG02CTC showing higher expression of EGFR, FGFR2, receptor tyrosine-protein kinase erbB-2 (ERBB2), and Janus kinase/signal transducer and activator of transcription (JAK/STAT) pathway genes compared to UWG01CTC, while UWG01CTC having marked expression of the *DLL3 *gene, this study highlights potential druggable targets whose activity should be explored in order to define new personalized types of treatment^[22]^. Finally, since UWG01CTC displayed lower expression of DNA damage response enzymes than UWG02CTC, it has also been noted that a synergistic effect of radiotherapy when combined with carboplatin for UWG01CTC line^[22] ^[Table 2].

Later in 2020, Lee *et al.*^[23] ^succeeded in expanding *ex vivo *CTCs from SCLC patients. For this study, a new system was developed to culture CTCs, involving the implementation of a biomimetic material called binary colloidal crystal^[23]^. With the use of binary colloidal crystal, it was possible to build a suitable surface for CTCs expansion, and it was also found that CTC from SCLC formed spheroids, which were observed after 14 days and continued to grow, showing to be still viable after 40 days^[23]^. During this period, drug sensitivity tests were performed on these CTC cultures to evaluate the response to standard first-line treatment of SCLC, which basically consists of a platinum doublet: cisplatin or carboplatin combined with etoposide^[23]^. This work demonstrated that CTC cultures recapitulate their originating patients’ outcomes, thus highlighting the possibility for expansions of CTCs to predict responses to therapy^[23]^. Since PD-L1 expression has been detected through immunofluorescence-based analyses, this study also suggests exploring the efficacy of immune checkpoint blockade, which are emerging as a valid treatment for SCLC patients^[23] ^[Table 2].

## CONCLUSIONS

*Ex vivo *expansions of CTCs clearly represent a potential tool to examine tumor characteristics, unraveling new biomarkers as well as possibly predicting drug sensitivity, eventually leading to optimized and personalized treatment strategies. However, the establishment of CTC lines is still tremendously challenging and currently not capable of informing clinical decisions.

One of the most important limiting factors is the timing of the process, which is not rapid enough to guide therapeutic choices for donor patients^[3,83]^. Indeed, the establishment of CTC lines and their consequent stabilization generally require months to be obtained^[3,12]^. In this context, several techniques are currently under investigation, such as innovative culture media or support surfaces capable of promoting rapid cell growth and survival^[3]^.

Secondly, since being an essential prerequisite to successfully yield cell lines from CTCs, high CTC counts (> 300 CTCs) constitute another important obstacle that restricts the use of these models in patients with the advanced-stage disease^[83]^. Novel applications of diagnostic leukapheresis^[85] ^or *in vivo *CTC capture devices^[86,87]^, allowing the isolation of higher numbers of CTCs than conventional enrichment methods, are expected to be a solution for this impediment.

Furthermore, experimental studies suggested that < 0.01% of cancer cells are supposed to initiate metastasis^[88-90]^. The low frequency of metastatic-inducing CTC among highly heterogeneous populations of tumor cells released in the circulation must be considered particularly when performing drug sensitivity or drug screening tests for clinical purposes^[32]^*. *Indeed, as Hamilton *et al.*^[20] ^demonstrated, CTCs may not reflect treatment responses of primary and metastatic lesions. Moreover, it is crucially important to assess whether the analyses conducted on a cell population that basically derives from few CTCs can be significantly representative of the entire tumor complexity^[3]^.

In conclusion, despite being extremely promising, the use of CTC lines, which will hopefully fulfill soon the great expectation of providing the exact information to offer the best therapeutic option possible to cancer patients, still requires further optimizations to allow translation into the clinic.

## DECLARATIONS

### Copyright

© The Author(s) 2022.
